# Tensile properties of human spinal dura mater and pericranium

**DOI:** 10.1007/s10856-022-06704-0

**Published:** 2022-12-31

**Authors:** Sacha Cavelier, Ryan D. Quarrington, Claire F. Jones

**Affiliations:** 1 Adelaide Spinal Research Group, Centre for Orthopaedic & Trauma Research, Adelaide Medical School, The University of Adelaide, Adelaide, SA 5005 Australia; 2 Department of Mechanical Engineering, McGill University, Montréal, QC H3A 0C3 Canada; 3 School of Mechanical Engineering, The University of Adelaide, Adelaide, SA 5005 Australia; 4 Department of Orthopaedics and Trauma, Royal Adelaide Hospital, Adelaide, SA 5000 Australia

## Abstract

Autologous pericranium is a promising dural graft material. An optimal graft should exhibit similar mechanical properties to the native dura, but the mechanical properties of human pericranium have not been characterized, and studies of the biomechanical performance of human spinal dura are limited. The primary aim of this study was to measure the tensile structural and material properties of the pericranium, in the longitudinal and circumferential directions, and of the dura in each spinal region (cervical, thoracic and lumbar) and in three directions (longitudinal anterior and posterior, and circumferential). The secondary aim was to determine corresponding constitutive stress–strain equations using a one-term Ogden model. A total of 146 specimens were tested from 7 cadavers. Linear regression models assessed the effect of tissue type, region, and orientation on the structural and material properties. Pericranium was isotropic, while spinal dura was anisotropic with higher stiffness and strength in the longitudinal than the circumferential direction. Pericranium had lower strength and modulus than spinal dura across all regions in the longitudinal direction but was stronger and stiffer than dura in the circumferential direction. Spinal dura and pericranium had similar strain at peak force, toe, and yield, across all regions and directions. Human pericranium exhibits isotropic mechanical behavior that lies between that of the longitudinal and circumferential spinal dura. Further studies are required to determine if pericranium grafts behave like native dura under in vivo loading conditions. The Ogden parameters reported may be used for computational modeling of the central nervous system.

**Figure:**
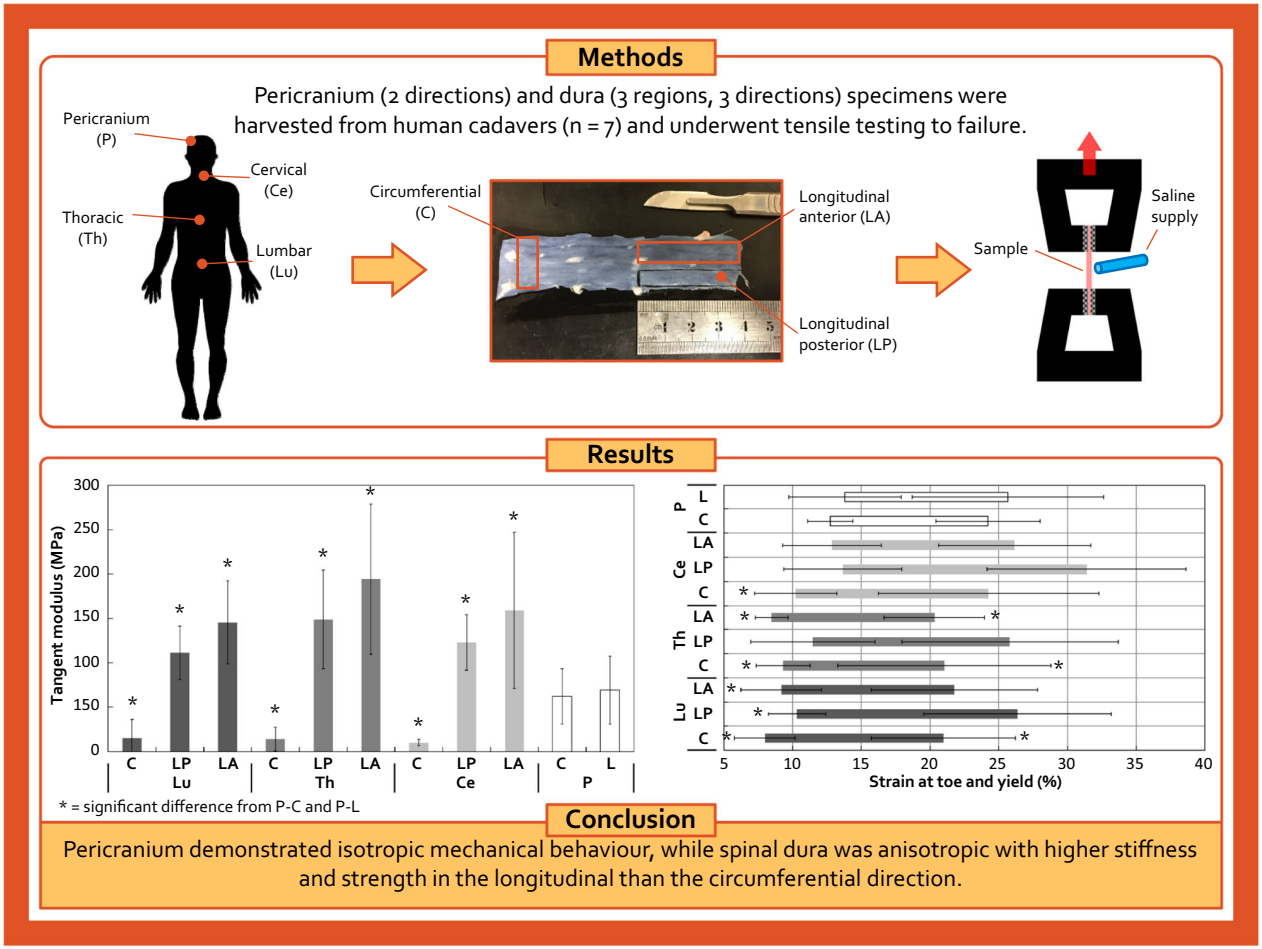
Graphical abstract

Graphical abstract

## Introduction

Spinal duraplasty is a surgical procedure in which the dura is grafted to provide a watertight closure of the dural sac, in an effort to reduce the risk of infection, prevent cerebrospinal fluid leakage and inflow of blood and contaminants [1–3], and potentially decompress the spinal cord [4, 5]. Spinal duraplasty may be required to repair incidental dural tears [6–8], following intradural tumor resection [9–12], or to expand the dura to decompress the spinal cord following trauma [4, 5] or myelopathy [13, 14]. Graft performance is likely to be optimal when the mechanical and structural properties are similar to that of native dura, while the material must also be suturable and biocompatible [15]. Various synthetic materials [16–20] and harvested autologous membranes [15, 20–25] have been evaluated in the laboratory or clinically for cranial or spinal applications, but an ideal graft material has not been identified [26]. Pericranium has demonstrated clinical efficacy as a cranial and cranio-cervical dural graft [24, 25, 27–32], but the material and mechanical properties of the pericranium, and their similarity to spinal dura mater, are not known.

Data describing the structural and material properties of human central nervous system tissues are required to develop accurate numerical and computational models of brain and spinal cord trauma [33] and to investigate cerebrospinal fluid system dynamics and physiology [34–38]. While finite element (FE) models may omit spinal dura or represent it as a linear elastic material [39–47], human and animal dura mater have a nonlinear stress–strain response that is characteristic of collagenous soft tissues [48, 49]. Few FE models have incorporated nonlinear elastic material properties for spinal dura [33, 50, 51], likely in part due to a lack of empirically derived nonlinear stress–strain constitutive models for human spinal dura; spinal dura remains under-investigated compared to cranial dura [52]. Mechanical characterization of human *cranial* dura has suggested that the stress–strain response *σ(ε)* is best approximated by a one-term Ogden hyperelastic model: $\sigma \left( \varepsilon \right) = \frac{{2G}}{\alpha }\left( {\left( {1 + \varepsilon } \right)^{\alpha - 1} - (1 + \varepsilon )^{ - 0.5\alpha - 1}} \right)$ where *G*_0_ is the instantaneous shear modulus [40]. An accurate characterization of the parameters *G*_0_ and *α* is therefore important to improve the constitutive equations of human spinal dura and pericranium for use in computational models.

The primary aim of this study was to characterize the tensile mechanical and material properties of human pericranium and spinal dura (cervical, thoracic, and lumbar) in the longitudinal and circumferential directions. This data was used to identify the regions of spinal dura with tensile properties that are most comparable to pericranium, and to determine the constitutive equations for each tissue region and direction.

## Methods

Institutional Human Research Ethics Committee approval was granted for this study (Reference No. H-2018-261). Sample preparation and mechanical testing protocols were adapted from those described previously for porcine dura and pericranium specimens [53].

### Sample preparation

Complete or partial spinal cords with intact dura, and/or posterior pericranium (50 × 50 mm) were harvested from seven human cadavers (Table 1; mean age = 82 years [range 66–96], 4 male). Spinal cords were immediately separated into cervical (Ce), thoracic (Th), and lumbar (Lu) regions. After harvesting, all specimens were wrapped in saline-soaked gauze, double bagged, and frozen at −20 °C. Prior to preparation, specimens were defrosted at 4 °C for 15 h. The spinal arachnoid–dura mater bilayer was sectioned into 36 ± 2 × 5 ± 1 mm rectangular samples in three orientations: longitudinal anterior (LA), longitudinal posterior (LP), and circumferential (C) (Table 1). Pericranium samples were prepared similarly, in two orientations: longitudinal (P-L) and circumferential (P-C) (Table 1). Hydration was maintained by spraying the samples with phosphate-buffered saline (PBS) throughout the preparation.

**Table 1:** Donor demographics, specimen designation, number of samples, and mean thickness, for each region and direction

Donor			Lumbar			Thoracic			Cervical			Pericranium	
ID	Age	Sex	C	LP	LA	C	LP	LA	C	LP	LA	C	L
H014	87	M	–	–	–	–	–	–	–	–	–	5	5
H015	78	M	–	–	–	–	–	–	–	–	–	4	4
H021	66	F	5	4	4	4	4	4	4	4	5	4	4
H023	83	F	2	2	2	2	4	3	2	2	2	4	4
H025	96	F	3	3	3	4	4	4	3	3	3	4	4
H038	81	M	–	–	–	3	3	5	–	–	–	–	–
H042	80	M	–	–	–	4	4	4	–	–	–	–	–
Total			10	9	9	17	19	19	9	9	10	21	21
Mean thickness (SD) [μm]:			159 (44)	171 (48)	183 (43)	224 (79)	261 (82)	147 (68)	264 (84)	225 (89)	213 (96)	131 (39)	157 (49)

*C* circumferential, *L* longitudinal, *A* anterior, *P* posterior

Dashes indicates that this tissue was not available for this donor

Cyanoacrylate adhesive (Loctite, Henkel, Düsseldorf, Germany) fixed 7 × 10 mm sandpaper rectangles to the dried distal ends of each sample to prevent slippage during tensile loading [54]. The average sample thickness was calculated from three-micrometer measurements (293-330-30, Mitutoyo, Sakado, Japan; resolution 1 μm, accuracy ±2 μm) at the center and at each end of the unloaded sample. Samples were stored on PBS-soaked gauze in a sealed container at 4 °C for 1 – 4 h until testing was performed in a temperature-controlled room (22 °C).

### Mechanical testing

Samples were fixed within the test space of a uniaxial materials testing machine (5543, Instron, High Wycombe, UK) using pneumatic side-action grips (5 bar). A 50 N load cell (2530-50N, Instron, High Wycombe, UK; linearity ±0.25%) and a linear variable differential transformer (73-10-10, Instron, High Wycombe, UK; resolution 0.001 mm, accuracy 0.25%) recorded axial force and actuator position, respectively, at 10 Hz. The load cell was zeroed with the sample suspended from the upper clamp to account for the weight of the specimen, prior to attaching the lower clamp. Samples were then pre-loaded to 0.05 N and Vernier calipers (500-196-30, Mitutoyo, Sakado, Japan; resolution 0.01 mm) were used to measure the width at the mid-span. For preconditioning [55], each sample underwent three cycles of sub-yield tensile loading at 1.6 mm/min. A pilot study (Supplementary Material A) determined the ideal number of load-unload cycles, and showed that yield point was specific to the anatomical region of the sample. Three preconditioning strain limits (approximately the middle of the linear region) were identified: 10% for thoracic and lumbar dura; 15% for pericranium; and, 20% for cervical dura. Following preconditioning, samples were loaded in tension at 1.6 mm/min until failure occurred. Sample hydration was maintained throughout via a continuous flow of room-temperature phosphate-buffered saline (22 °C).

### Data analysis

Data analysis was performed using custom Matlab code (R2015a, Mathworks, Massachusetts, USA). Raw data were filtered with a second-order, two-way Butterworth low-pass filter (cut-off frequency of 10 Hz). Engineering stress $\left( {\sigma = \frac{F}{{wt}}} \right)$ and engineering strain $\left( {\varepsilon = \frac{d}{l}} \right)$ were derived from the displacement *d*, the load *F*, the width of the sample *w*, its initial length *l*, and its thickness, *t*. The peak stress and the linear region of the stress–strain curve were automatically detected [53], from which the strain at peak, elastic modulus (slope of the linear region), and strains at the lower (toe) and upper (yield) bounds of the linear region were determined (Supplementary Material B). Structural properties (peak force, extension at peak, stiffness, extension at toe/yield) were similarly extracted. A one-term Ogden hyperelastic model $\sigma \left( \varepsilon \right) = \frac{{2G_0}}{\alpha }\left( {\left( {1 + \varepsilon } \right)^{\alpha - 1} - \left( {1 + \varepsilon } \right)^{ - 0.5\alpha - 1}} \right)$ was fitted to the filtered stress–strain curves (Supplementary Fig. S2). The model accommodated the low stiffness nonlinear region (toe region) and the higher stiffness linear region which provided the parameters of the fit (*α* and *G*_0_, the shear modulus). The yield region of the specimen response was excluded because its exponential form is not accommodated by the Ogden model.

For each structural and material property (ten outcomes in total), linear regression models with cluster-robust standard errors were used to assess if the property differed due to region (cervical, thoracic, and lumbar spinal dura, or pericranium) and/or orientation (C, L, LA, or LP), using SPSS v26 (IBM, Illinois, USA). Multiple samples were obtained from the same region of some donors, so cluster-robust standard errors accounted for non-independent observations. Sequential Bonferroni-adjusted post hoc analyses provided *p* values for all region-orientation comparisons. A threshold of significance of 0.05 was chosen to reject the hypothesis that a spinal region-orientation was similar to pericranium.

## Results

A total of 112 spinal dura samples from 5 donors and 42 pericranium samples from 5 donors were tested (Table 1). Of these, eight samples were excluded from the study because failure occurred near the grip edge. No sample failed during preconditioning. Spinal dura from the cervical region was thicker than lumbar (*p* < 0.01), but similar to thoracic (*p* = 0.28), dura. Pericranium was thinner than dura from any region (*p* < 0.001). Large standard deviations indicated non-uniformity of tissue thickness and differences between regions were not significant.

**Fig. 1:**
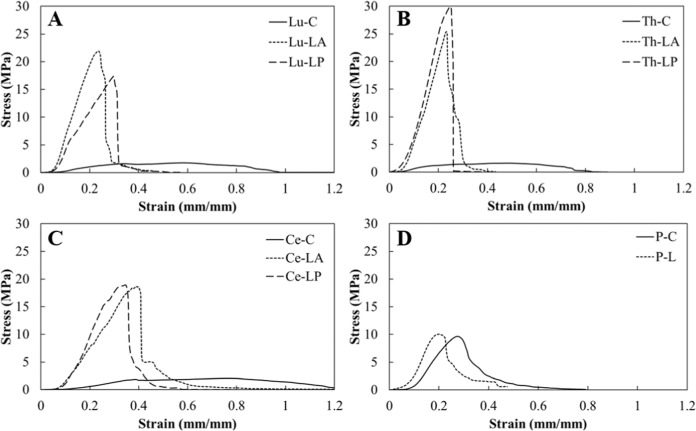
Typical stress–strain curves for the lumbar (**A**; Lu), thoracic (**B**; Th), and cervical (**C**; Ce) spinal dura regions, and for pericranium (**D**; P). C circumferential, L longitudinal, A anterior, P posterior

**Fig. 2:**
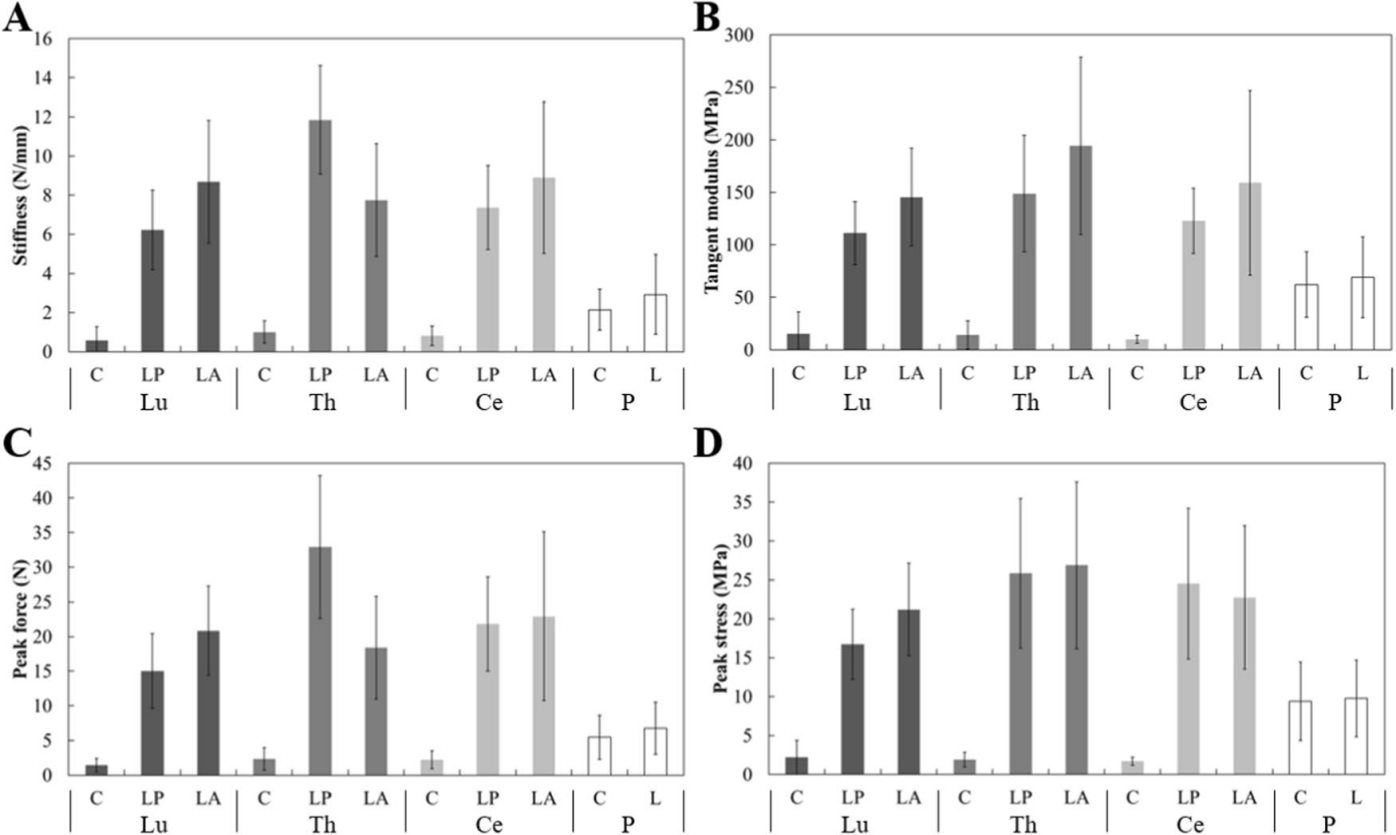
Mean and standard deviation of **A** stiffness; **B** tangent modulus; **C** peak force and **D** strength for spinal dura in lumbar (Lu), thoracic (Th), and cervical (Ce) regions, and pericranium (P). For all outcomes, the spinal region*orientation groups were significantly different to both circumferential and longitudinal pericranium. C circumferential, L longitudinal, A anterior, P posterior

**Fig. 3:**
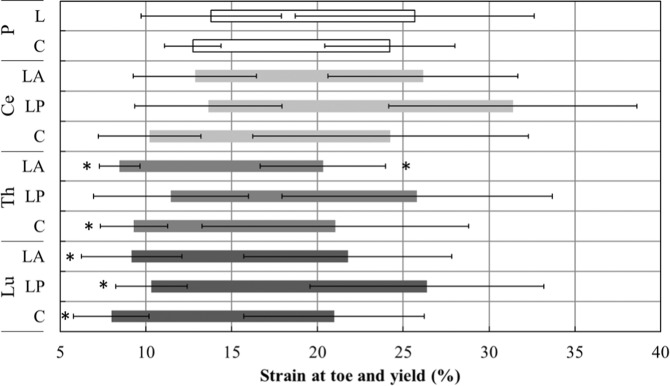
Quasi-linear elastic region (shaded bars) for the lumbar (Lu), thoracic (Th), and cervical (Ce) spinal dura, and pericranium (P) samples. Mean and standard deviation of strain at toe and yield are shown. * indicates a significant difference with P-L and P-C. C circumferential, L longitudinal, A anterior, P posterior

The pericranium and longitudinal spinal dura samples from each region typically displayed J-shaped stress–strain curves consistent with other collagenous biological soft tissues, comprising a toe region, quasi-linear elastic region, yield, and abrupt failure characterized by a sharp stress reduction (Fig. 1). In contrast, the circumferential spinal dura samples exhibited prolonged plastic deformation without abrupt failure. Pericranium was isotropic, with no observed differences in material or structural properties between circumferential and longitudinal samples (Figs. 1D, 2, and 3).

For all spinal regions, in the longitudinal orientation, spinal dura demonstrated greater stiffness, tangent modulus, peak force, and peak stress than pericranium, while in the circumferential orientation these parameters were all significantly lower for spinal dura than for pericranium (Table 2 and Fig. 2). Pericranium had comparable extension and strain, at yield and peak stress, to LP spinal dura from all spinal regions (except yield strain for Ce-LP), and to LA for cervical and lumbar dura (Table 2 and Fig. 3). Thoracic LP and cervical LA dura were similar to pericranium for all extension and strain outcomes.

**Table 2:** *P* values for comparisons between each dura specimen type, and circumferential and longitudinal pericranium, for the ten outcome measures

Outcomes		Lumbar			Thoracic			Cervical		
		C	LP	LA	C	LP	LA	C	LP	LA
Stiffness	P-C	**<0.01**	**<0.01**	**<0.01**	**<0.01**	**<0.01**	**<0.01**	**<0.01**	**<0.01**	**<0.01**
	P-L	**<0.01**	**<0.01**	**<0.01**	**<0.01**	**<0.01**	**<0.01**	**<0.01**	**<0.01**	**<0.01**
Peak force	P-C	**<0.01**	**<0.01**	**<0.01**	**<0.01**	**<0.01**	**<0.01**	**<0.01**	**<0.01**	**<0.01**
	P-L	**<0.01**	**<0.01**	**<0.01**	**<0.01**	**<0.01**	**<0.01**	**<0.01**	**<0.01**	**<0.01**
Extension at peak	P-C	**0.02**	0.94	0.16	1.00	0.94	**<0.01**	0.94	0.79	1.00
	P-L	**0.03**	0.60	0.09	1.00	0.55	**<0.01**	1.00	1.00	1.00
Extension at toe	P-C	**<0.01**	**<0.01**	**<0.01**	**<0.01**	0.24	**<0.01**	**<0.01**	0.89	0.97
	P-L	**<0.01**	**<0.01**	**<0.01**	**<0.01**	0.11	**<0.01**	**<0.01**	0.59	0.48
Extension at yield	P-C	**<0.01**	1.00	0.10	**<0.01**	0.88	**<0.01**	0.88	0.71	1.00
	P-L	**0.01**	1.00	0.09	**<0.01**	0.63	**0.01**	0.61	1.00	1.00
Tangent modulus	P-C	**<0.01**	**<0.01**	**<0.01**	**<0.01**	**<0.01**	**<0.01**	**<0.01**	**<0.01**	**<0.01**
	P-L	**<0.01**	**<0.01**	**<0.01**	**<0.01**	**<0.01**	**<0.01**	**<0.01**	**<0.01**	**<0.01**
Peak stress	P-C	**<0.01**	**<0.01**	**<0.01**	**<0.01**	**<0.01**	**<0.01**	**<0.01**	**<0.01**	**<0.01**
	P-L	**<0.01**	**<0.01**	**<0.01**	**<0.01**	**<0.01**	**<0.01**	**<0.01**	**<0.01**	**<0.01**
Strain at peak	P-C	**0.02**	1.00	0.52	**0.04**	1.00	**<0.01**	0.27	0.07	1.00
	P-L	**0.03**	1.00	0.31	0.31	1.00	**<0.01**	0.66	0.66	1.00
Strain at toe	P-C	**<0.01**	**<0.01**	**<0.01**	**<0.01**	1.00	**<0.01**	0.06	1.00	1.00
	P-L	**<0.01**	**<0.01**	**<0.01**	**<0.01**	0.35	**<0.01**	**0.03**	1.00	1.00
Strain at yield	P-C	0.64	1.00	1.00	0.80	1.00	**0.01**	1.00	**0.03**	1.00
	P-L	0.31	1.00	0.64	0.35	1.00	**0.02**	1.00	0.31	1.00

*P* Pericranium, *C* circumferential, *L* longitudinal, *A* anterior, *P* posterior

Significant differences in bold (*α* = 0.05)

Toe and linear regions of stress–strain curves were fit to a one-term Ogden model. The *G*_0_ and *α* parameters for the Ogden models from each tissue region and orientation are provided (Table 3).

**Table 3:** Mean values, standard deviations and 95% confidence intervals for the Ogden parameters *G*_0_ and *α* of each specimen group

Group	*G*_0_ (MPa)			*α*		
	Mean	SD	95% CI	Mean	SD	95% CI
Lu-C	0.71	0.45	0.64–0.78	19.84	7.70	14.81–24.87
Lu-LP	4.85	2.68	4.45–5.25	22.20	6.72	17.81–26.59
Lu-LA	4.96	1.52	4.74–5.18	28.97	9.07	23.05–34.90
Th-C	0.44	0.19	0.42–0.47	27.15	8.97	22.89–31.42
Th-LP	5.16	2.61	4.84–5.47	25.81	10.39	20.87–30.75
Th-LA	6.77	4.07	6.11–7.43	32.18	9.75	27.54–36.81
Ce-C	0.53	0.21	0.50–0.55	21.24	7.92	16.06–26.42
Ce-LP	4.53	3.79	4.03–5.02	20.99	9.91	14.52–27.47
Ce-LA	2.58	0.92	2.46–2.71	25.22	7.68	20.20–30.23
P-C	0.87	0.58	0.77–0.97	27.87	4.80	25.76–29.97
P-L	0.86	0.68	0.75–0.97	29.00	6.21	26.34–31.66

*Lu* lumbar, *Th* thoracic, *Ce* cervical, *P* pericranium, *C* circumferential, *L* longitudinal, *A* anterior, *P* posterior

## Discussion

Because of their biological and biomechanical performance, autologous membranes are commonly used in duraplasty procedures [15, 21–23, 31, 56]. For example, periosteum has reported benefits of non-immunogenicity, nontoxicity, ability to be sutured watertight, rapid biological/tissue integration, low cost, and low risk of infection in human studies [1, 24, 25, 28, 29, 31]. Pericranium has been identified as a promising autologous cranio-spinal dura graft material [24, 25]. This study characterized and compared the mechanical and material properties of human pericranium and spinal dura tissue. Tensile mechanical tests were performed on human cadaveric spinal dura, and pericranium, in the longitudinal and circumferential directions. Pericranium was isotropic and exhibited similar extension and strain at yield and failure to longitudinal dura tissue, but the ultimate stress and tangent modulus were significantly lower than that of longitudinal dura.

Anisotropic behavior of human spinal dura was observed, with peak stress and modulus consistently largest in the longitudinal direction. This behavior has been described for lumbar spinal dura [57, 58] and attributed to the alignment of collagen fibers in the longitudinal direction [58]. For lumbar dura (donor age 39–86 years, mixed anterior/posterior), Runza et al. reported Young’s moduli of 42–144 MPa (longitudinal) and 4–6 MPa (circumferential), and ultimate tensile strength of 8–20.2 MPa (longitudinal) and 3–4.5 MPa (circumferential). The latter were similar to that obtained for lumbar dura in the current study (Fig. 2D), and the former were similar for the longitudinal posterior orientation but smaller in the longitudinal anterior and circumferential orientations (Fig. 2B). Tencer et al. [59] reported longitudinal (mixed anterior/posterior) apparent modulus similar to that observed for thoracic posterior and cervical posterior regions, but higher than that observed in lumbar region, in the current study. The trend was similar for the peak stress. Previous reports of strain at peak force for the longitudinal orientation range from 20 to 62% [57, 58] compared to 27 ± 6% in the current study. Substantial variation in strain at peak force for the circumferential orientation (83 ± 54%) was observed in the current study, consistent with previous reports [57–59], and may be due to local differences in tissue microstructure [58].

There is limited reported data with which to compare the pericranium mechanical properties. The ultimate strength of pericranium in this study (9.41 (C) to 9.76 (L) MPa) was higher than that reported for periosteum in the nasal region (3.66–4.04 MPa) [60]. Similarly, the tangent modulus of pericranium in this study (62.0 (C)–69.2 (L) MPa) was greater than that of mandibular periosteum (8.0–16.4 MPa) [61], which may be due to the different region of harvest, as well as different testing protocols.

The stiffness, modulus, peak force, and peak stress of pericranium were greater than that of circumferential spinal dura and lower than that of longitudinal dura. However, strains in the linear region and at peak were mostly similar for pericranium and all regions and orientations of spinal dura. A similar study comparing porcine pericranium and spinal dura reported similar stiffness and peak stress, but different linear region and peak strains, between these tissues [53].

The lower strength of pericranium, than longitudinal spinal dura, may limit its ability to protect the dural sac as a graft material post surgery. However, its lower stiffness may be beneficial for cranial decompression following traumatic brain injury. For instance, in order to close the cranial dura after decompressive craniectomy, pericranium is preferred by neurosurgeons over rigid allografts or synthetic dural substitutes because of its ability to stretch and allow the brain to herniate [62]. The relatively low stiffness of pericranium may also be advantageous in the case of traumatic spinal cord injury with oedemic-related cord-dura contact, where duraplasty may be indicated in some patients to decompress the spinal cord [4, 63]. However, the potential influence of dural graft material properties on local CSF dynamics is unknown [35]. The structural and material properties of pericranium were more similar to spinal dura than most of the synthetic biomaterials used for duraplasty [64–66]. The strength (9.41 (C) to 9.76 (L) MPa) was higher than that of Tutopatch® bovine pericardium (3.51 MPa) and its modulus (62.0–69.2 MPa) was higher than that of Gore-Tex® Expanded Cardiovascular Patch (18.26 MPa) [64]. The current study also suggests the strain response of pericranium is also more compatible with spinal dura. For example, a linear region corresponding to 40–60% of strain is reported for Durepair® and 0.5–1.5% for Gore-Tex® Expanded Cardiovascular Patch [64] while in the current study the strain at yield was 24.2–25.7% for pericranium and 20.3–31.4% for spinal dura.

While some manufactured materials that have been used as dural grafts have some mechanical similitude to native dura [64, 66], none have the combination of strength, modulus, and strain at peak force measured in the current study for spinal dura and pericranium (Table 4). In addition, some synthetic grafts have demonstrated an increased risk of infection compared to autologous pericranium grafts [24] and have exhibited limited tissue integration [67, 68]. In some studies, non-autologous dural grafts have been associated with complications such as hemorrhage, bacteria and virus transmission, Creutzfeldt-Jakob disease transmission, immune response, slower healing, or graft dissolution [19, 25, 69–72].

**Table 4:** Mechanical properties previously reported for human spinal dura mater and manufactured materials used as dura grafts

Study	Region	Properties		
		Strength (MPa)	Strain at failure (%)	Elastic modulus (MPa)
Tencer et al. (1985) [59]^a^	Lumbar	L: 29	L: 27	L: 172
	Thoracic	L: 28	L: 37	L: 150
	Cervical	L: 26	L: 34	L: 129
Zarzur et al. (1996) [57]^b^	Lumbar	N/A	C: 45.1 ± 11.5	N/A
			L: 39.7 ± 12.4	
Runza et al. (1999) [58]^a^	Lumbar	C: 3–4.5	C: 38–48	C: 4–6
		L: 8–20.2	L: 20–62	L: 42–144
Present study	Lumbar	C: 2.21 ± 2.17	C: 82.50 ± 54.47	C: 15.11 ± 21.16
		LP: 16.73 ± 4.51	LP: 28.28 ± 7.30	LP: 111.12 ± 29.91
		LA: 21.19 ± 5.96	LA: 26.28 ± 5.22	LA: 145.43 ± 46.74
	Thoracic	C: 1.91 ± 0.95	C: 38.93 ± 13.04	C: 14.18 ± 13.62
		LP: 25.86 ± 9.62	LP: 29.54 ± 7.32	LP: 148.76 ± 55.65
		LA: 26.88 ± 10.73	LA: 22.91 ± 3.87	LA: 194.24 ± 85.53
	Cervical	C: 1.71 ± 0.51	C: 40.41 ± 16.61	C: 10.13 ± 3.77
		LP: 24.52 ± 9.68	LP: 35.83 ± 6.54	LP: 122.86 ± 31.00
		LA: 22.74 ± 9.19	LA: 29.90 ± 5.35	LA: 159.05 ± 88.00
	Pericranium	C: 9.41 ± 5.02	C: 29.62 ± 5.73	C: 62.00 ± 31.15
		L: 9.76 ± 4.94	L: 31.52 ± 9.09	L: 69.21 ± 38.44
Gore-Tex® Expanded Cardiovascular Patch [64]		22.03 ± 0.60	0.5–1.5^a^	18.26 ± 8.45
Dura-Guard® [66]		13.50 ± 3.34		81.33 ± 20.48
Tutopatch® [59]		3.51 ± 0.63	10–30^a^	N/A^c^
Durepair® [64] and [66]		19.59 ± 0.65	50–70^a^	54.16 ± 4.82
		22.70 ± 2.83	–	69.94 ± 9.49
DuraGen® [66]		N/A^c^	N/A^c^	N/A^c^

Ranges, mean values or standard deviations are represented when available

*C* circumferential, *L* longitudinal, *A* anterior, *P* posterior

^a^Values are ranges taken from graphs

^b^Elastic moduli reported in Zarzur et al. (1996) are not provided herein because of an apparent error in the Modulus of Elasticity equation provided in that paper, and therefore uncertainty in the results

^c^Properties could not be calculated/measured due to low tensile strength

The hyperelastic Ogden model parameters provided for the elastic regions of the pericranium and spinal dura stress–strain data may be used to improve future numerical analyses that model human spinal dura and pericranium. In part due to a lack of appropriate data, most reported computational models of the spine apply simple linear elastic properties to the dura [34–47]. As is typical in the application of Ogden models to hyperelastic collagenous tissue, the exponential form of the model does not account for the yield region in which sequential failure of collagen fibers occurs [48, 73, 74], thus the derived material models are limited to simulating mechanics below the damage threshold. Values for *G*_0_ were more distributed than *α*, as evidenced by relatively large standard deviations.

Some limitations accompany this study. There were relatively wide distributions for some outcomes (particularly extension and strain at toe/yield); however, this is not unique to this study, for example, similar variability of the shear modulus (*G*_0_) has been reported for human cranial dura [40], and animal spinal [48, 73, 75] and cranial dura [39, 74]. This spread of data may be partly attributed to specimen-specific factors such as age, health conditions, physiology, and cause of death; for instance, the tensile properties of human cranial dura (elastic modulus, tensile strength and maximum strain) are inversely related to donor age [76]. Other extrinsic factors such as tissue storage and preparation conditions may also affect the mechanical properties [77]. Dura is a composite material with heterogeneous and site-specific microstructural arrangements and variable geometry [58] which contributes to variation in mechanical behavior. The overall effect of these intrinsic and extrinsic factors could be somewhat mitigated by increasing the number of donors, collecting samples from every region of interest in each donor, and increasing the number of samples tested from each region.

The experimental procedures were developed to minimize damage to the tissues during sample preparation, and to prevent sample slippage during testing. A combination of sandpaper, cyanoacrylate adhesive, and pneumatic grips was used to minimize the risk of slippage [48, 54, 74]. In addition, rectangular, rather than dumbbell-shaped, samples were used to reduce the damage to the tissue during preparation. Such rectangular geometry is known to underestimate the stress due to stress concentration at the grip [78]. The largest span possible was used to increase the specimen length-to-width ratio and minimize the stress concentration effect, and to maintain a uniform stress far away from the gripped ends [78]. The span length and width were limited by the circumference of the spinal dura and the continuity of defect-free zones (i.e. absence of nerve roots or damage during tissue harvest), respectively. Only eight samples were discarded because failure occurred near the grip; however, digital image correlation could be used to improve slip detection and mid-span strain measurements.

This study describes the uniaxial tensile mechanical response of human pericranium and spinal dura. Pericranium exhibited isotropic mechanical behavior, and demonstrated similar strains at the boundaries of the linear region, and at peak force, to the anisotropic spinal dura from all spinal regions. Pericranium (in both tested directions) had lower strength and stiffness than spinal dura in the longitudinal orientation, but had higher strength and stiffness than spinal dura in the circumferential direction. Taken together, these findings suggest that pericranium may have suitable extensibility when used as a spinal dural graft material, but further investigations are required to assess its protective ability during physiological loading.

## Supplementary information

Supplementary Materials

## Data Availability

The datasets used and/or analyzed during the current study are available from the corresponding author upon reasonable request.

## References

[1] Kosnik EJ (1998). Use of ligamentum nuchae graft for dural closure in posterior fossa surgery. Technical note. J Neurosurg.

[2] Von Wild KR (1999). Examination of the safety and efficacy of an absorbable dura mater substitute (dura patch) in normal applications in neurosurgery. Surgical Neurol.

[3] Williams LE, Vannemreddy PS, Watson KS, Slavin KV (2013). The need in dural graft suturing in Chiari I malformation decompression: a prospective, single-blind, randomized trial comparing sutured and sutureless duraplasty materials. Surgical Neurol Int.

[4] Phang I, Werndle MC, Saadoun S, Varsos G, Czosnyka M, Zoumprouli A, Papadopoulos MC (2015). Expansion duroplasty improves intraspinal pressure, spinal cord perfusion pressure, and vascular pressure reactivity index in patients with traumatic spinal cord injury: injured spinal cord pressure evaluation study. J Neurotrauma.

[5] Zhu FZ, Yao S, Ren ZW, Telemacque D, Qu YZ, Chen KF, Yang F, Zeng L, Guo XD (2019). Early durotomy with duroplasty for severe adult spinal cord injury without radiographic abnormality: a novel concept and method of surgical decompression. Eur Spine J.

[6] Dafford EE, Anderson PA (2015). Comparison of dural repair techniques. Spine J.

[7] Wolff S, Kheirredine W, Riouallon G (2012). Surgical dural tears: prevalence and updated management protocol based on 1359 lumbar vertebra interventions. Orthop Traumatol Sur.

[8] Taylor C, Khan A, Shenouda E, Brooke N, Nader-Sepahi A (2022). Dural tear repair surgery comparative analysis: a stitch in time saves nine. Eur Spine J.

[9] Arnautovic KI, Kovacevic M (2016). Csf-related complications after intradural spinal tumor surgery: utility of an autologous fat graft. Med Arch.

[10] Ito K, Aoyama T, Nakamura T, Hanaoka Y, Horiuchi T, Hongo K (2016). Novel dural incision and closure procedure for preventing postoperative cerebrospinal fluid leakage during the surgical removal of dumbbell-shaped spinal tumors: technical note. J Neurosurg Spine.

[11] Sugawara T, Itoh Y, Hirano Y, Higashiyama N, Shimada Y, Kinouchi H, Mizoi K (2005). Novel dural closure technique using polyglactin acid sheet prevents cerebrospinal fluid leakage after spinal surgery. Neurosurgery.

[12] Masuda S, Fujibayashi S, Otsuki B, Kimura H, Neo M, Matsuda S (2016). The dural repair using the combination of polyglycolic acid mesh and fibrin glue and postoperative management in spine surgery. J Orthop Sci.

[13] Thakar S, Arun AA, Rajagopal N, Aryan S, Mohan D, Vijayan JE, Hegde AS (2021). Outcomes after cervical duraplasty for monomelic amyotrophy (Hirayama disease): results of a case-control study of 60 patients. J Neurosci Rural Pract.

[14] Chen JC, Li YN, Wang TY, Gao J, Xu JC, Lai RL, Tan DH (2017). Comparison of posterior fossa decompression with and without duraplasty for the surgical treatment of Chiari malformation type I in adult patients a retrospective analysis of 103 patients. Medicine.

[15] Thammavaram KV, Benzel E, Kesterson L (1990). Fascia lata graft as a dural substitute in neurosurgery. South Med J.

[16] Brown MH, Grindlay JH, Craig W (1948). The use of polythene film as a dural substitute; an experimental and clinical study. Surg Gynecol Obstet.

[17] Bhatia S, Bergethon PR, Blease S, Kemper T, Rosiello A, Zimbardi GP, Franzblau C, Spatz EL (1995). A synthetic dural prosthesis constructed from hydroxyethylmethacrylate hydrogels. J Neurosurg.

[18] Maurer PK, Mcdonald JV (1985). Vicryl (polyglactin 910) mesh as a dural substitute. J Neurosurg.

[19] Yamada K, Miyamoto S, Nagata I, Kikuchi H, Ikada Y, Iwata H, Yamamoto K (1997). Development of a dural substitute from synthetic bioabsorbable polymers. J Neurosurg.

[20] Finn MA, Faulkner ND, Hetzel SJ, Anderson PA (2011). Spinal duraplasty materials and hydrostasis: a biomechanical study laboratory investigation. J Neurosurg Spine.

[21] Gök A, Zorludemir S, Polat S, Tap Ö, Kaya M (1995). Experimental evaluation of peritoneum and pericardium as dural substitutes. Res Exp Med.

[22] Pařízek J, Měřička P, Špaček J, Němeček S, Eliáš P, Šercl M (1989). Xenogeneic pericardium as a dural substitute in reconstruction of suboccipital dura mater in children. J Neurosurg.

[23] O’neill P, Booth AE (1984). Use of porcine dermis as a dural substitute in 72 patients. J Neurosurg.

[24] Malliti M, Page P, Gury C, Chomette E, Nataf F, Roux F-X (2004). Comparison of deep wound infection rates using a synthetic dural substitute (neuro-patch) or pericranium graft for dural closure: a clinical review of 1 year. Neurosurgery.

[25] Stevens EA, Powers AK, Sweasey TA, Tatter SB, Ojemann RG (2009). Simplified harvest of autologous pericranium for duraplasty in Chiari malformation Type I. Technical note. J Neurosurg Spine.

[26] Nagel SJ, Reddy CG, Frizon LA, Chardon MK, Holland M, Machado AG, Al E (2018). Spinal dura mater: biophysical characteristics relevant to medical device development. J Med Eng Technol.

[27] Edward JK (1998). Use of ligamentum nuchae graft for dural closure in posterior fossa surgery. J Neurosurg.

[28] Lam FC, Kasper E (2012). Augmented autologous pericranium duraplasty in 100 posterior fossa surgeries—a retrospective case series. Neurosurgery.

[29] Mastronardi L, Cacciotti G, Caputi F, Roperto R, Tonelli MP, Carpineta E, Al E (2016). Underlay hourglass-shaped autologous pericranium duraplasty in “key-hole” retrosigmoid approach surgery: technical report. Surgical Neurol Int.

[30] Martinez-Lage J, Perez-Espejo MA, Palazon JH, Lopez Hernandez F, Puerta P (2006). Autologous tissues for dural grafting in children: a report of 56 cases. Childs Nerv Syst.

[31] Vanaclocha V, Saiz-Sapena N (1997). Duraplasty with freeze-dried cadaveric dura versus occipital pericranium for Chiari type I malformation: comparative study. Acta Neurochir (Wien).

[32] Perrini P (2015). Technical nuances of autologous pericranium harvesting for dural closure in Chiari malformation surgery. J Neurological Surg B Skull Base.

[33] Rycman A, Mclachlin S, Cronin DS (2022). Comparison of numerical methods for cerebrospinal fluid representation and fluid-structure interaction during transverse impact of a finite element spinal cord model. Int J Numer Meth Bio.

[34] Sweetman B, Linninger AA (2011). Cerebrospinal fluid flow dynamics in the central nervous system. Ann Biomed Eng.

[35] Martin BA, Reymond P, Novy J, Baledent O, Stergiopulos N (2012). A coupled hydrodynamic model of the cardiovascular and cerebrospinal fluid system. Am J Physiol Heart C.

[36] Cirovic S, Kim M (2012). A one-dimensional model of the spinal cerebrospinal-fluid compartment. J Biomech Eng.

[37] Bertram CD (2010). Evaluation by fluid/structure-interaction spinal-cord simulation of the effects of subarachnoid-space stenosis on an adjacent syrinx. J Biomech Eng.

[38] Bertram CD (2009). A numerical investigation of waves propagating in the spinal cord and subarachnoid space in the presence of a syrinx. J Fluid Struct.

[39] Macmanus DB, Pierrat B, Murphy JG, Gilchrist MD (2017). Protection of cortex by overlying meninges tissue during dynamic indentation of the adolescent brain. Acta Biomaterialia.

[40] De Kegel D, Vastmans J, Fehervary H, Depreitere B, Vander Sloten J, Famaey N (2018). Biomechanical characterization of human dura mater. J Mech Behav Biomed Mater.

[41] Bailly N, Diotalevi L, Beausejour MH, Wagnac E, Mac-Thiong JM, Petit Y (2020). Numerical investigation of the relative effect of disc bulging and ligamentum flavum hypertrophy on the mechanism of central cord syndrome. Clin Biomech.

[42] Diotalevi L, Bailly N, Wagnac E, Mac-Thiong JM, Goulet J, Petit Y (2020). Dynamics of spinal cord compression with different patterns of thoracolumbar burst fractures: Numerical simulations using finite element modelling. Clin Biomech.

[43] Henao J, Labelle H, Arnoux PJ, Aubin CE (2018). Biomechanical simulation of stresses and strains exerted on the spinal cord and nerves during scoliosis correction maneuvers. Spine Deform.

[44] Khuyagbaatar B, Kim K, Man Park W, Hyuk Kim Y. Biomechanical behaviors in three types of spinal cord injury mechanisms. J Biomech Eng. 2016;138. 10.1115/1.4033794. 10.1115/1.4033794 27276391

[45] Persson C, Summers J, Hall RM (2011). The importance of fluid-structure interaction in spinal trauma models. J Neurotrauma.

[46] Fradet L, Arnoux PJ, Callot V, Petit Y (2016). Geometrical variations in white and gray matter affect the biomechanics of spinal cord injuries more than the arachnoid space. Adv Mech Eng.

[47] Stoner KE, Abode-Iyamah KO, Fredericks DC, Viljoen S, Howard MA, Grosland NM (2020). A comprehensive finite element model of surgical treatment for cervical myelopathy. Clin Biomech.

[48] Maikos JT, Elias RaI, Shreiber DI (2008). Mechanical properties of dura mater from the rat brain and spinal cord. J Neurotrauma.

[49] Galford JE, Mcelhaney JH (1970). A viscoelastic study of scalp, brain, and dura. J Biomech.

[50] Jannesar S, Salegio EA, Beattie MS, Bresnahan JC, Sparrey CJ (2021). Correlating tissue mechanics and spinal cord injury: patient-specific finite element models of unilateral cervical contusion spinal cord injury in non-human primates. J Neurotrauma.

[51] Jannesar S, Nadler B, Sparrey CJ (2016). The transverse isotropy of spinal cord white matter under dynamic load. J Biomech Eng.

[52] Pearcy Q, Tomlinson J, Niestrawska JA, Mobius D, Zhang M, Zwirner J (2022). Systematic review and meta-analysis of the biomechanical properties of the human dura mater applicable in computational human head models. Biomech Model Mechanobiol.

[53] Cavelier S, Quarrington RD, Jones CF (2022). Mechanical properties of porcine spinal dura mater and pericranium. J Mech Behav Biomed Mater.

[54] Bilston LE, Thibault LE (1996). The mechanical properties of the human cervical spinal cord in vitro. Ann Biomed Eng.

[55] Fung Y-C (1993). Biomechanics: mechanical properties of living tissues.

[56] Rosen DS, Wollman R, Frim DM (2003). Recurrence of symptoms after Chiari decompression and duraplasty with nonautologous graft material. Pediatr Neurosurg.

[57] Zarzur E (1996). Mechanical properties of the human lumbar dura mater. Arq Neuropsiquiatr.

[58] Runza M, Pietrabissa R, Mantero S, Albani A, Quaglini V, Contro R (1999). Lumbar dura mater biomechanics: experimental characterization and scanning electron microscopy observations. Anesth Analg.

[59] Tencer AF, Allen BL, Ferguson RL (1985). A biomechanical study of thoracolumbar spine fractures with bone in the canal. Part III. Mechanical properties of the dura and its tethering ligaments. Spine (Philos Pa 1976).

[60] Zeng YJ, Sun XP, Yang J, Wu WH, Xu XH, Yan YP (2003). Mechanical properties of nasal fascia and periosteum. Clin Biomech (Bristol, Avon).

[61] Kadlub N, Debelmas A, Dallard J, Picard A, Boisson J (2020). Modeling of the human mandibular periosteum material properties and comparison with the calvarial periosteum. Biomech Model Mechanobiol.

[62] Cunan ET, Dudley R, Shemie SD. Decompressive craniectomy as a potentially reversible condition in brain death—brain stunning or skin and pericranium stretching? Can J Anaesth. 2022;69:811–4. 10.1007/s12630-022-02264-7. 10.1007/s12630-022-02264-7 35534771

[63] Aarabi B, Chixiang C, Simard JM, Chryssikos T, Stokum JA, Sansur CA, et al. Proposal of a management algorithm to predict the need for expansion duraplasty in American Spinal Injury Association Impairment Scale Grades A-C traumatic cervical spinal cord injury patients. J Neurotrauma. 2022. 10.1089/neu.2022.0218. 10.1089/neu.2022.0218 PMC9734016 35876459

[64] Kizmazoglu C, Aydin HE, Kaya I, Atar M, Husemoglu B, Kalemci O, Sozer G, Havitcioglu H (2019). Comparison of biomechanical properties of dura mater substitutes and cranial human dura mater: an in vitro study. J Korean Neurosurg Soc.

[65] Famaey N, Verhoeven J, Jacobs S, Pettinari M, Meyns B (2014). In situ evolution of the mechanical properties of stretchable and non-stretchable eptfe vascular grafts and adjacent native vessels. Int J Artif Organs.

[66] Zerris VA, James KS, Roberts JB, Bell E, Heilman CB. Repair of the dura mater with processed collagen devices. J Biomed Mater Res B Appl Biomater. 2007;83:580–8. 10.1002/jbm.b.30831. 10.1002/jbm.b.30831 17465025

[67] Collins RL, Christiansen D, Zazanis GA, Silver FH (1991). Use of collagen film as a dural substitute: preliminary animal studies. J Biomed Mater Res.

[68] Ellingsworth LR, Delustro F, Brennan JE, Sawamura S, Mcpherson J. The human immune response to reconstituted bovine collagen. J Immunol. 1986;136:877–82. 2416836

[69] Alleyne CH, Barrow DL (1994). Immune response in hosts with cadaveric dural grafts. Report of two cases. J Neurosurg.

[70] Thadani V, Penar PL, Partington J, Kalb R, Janssen R, Schonberger LB, Rabkin CS, Prichard JW (1988). Creutzfeldt-jakob disease probably acquired from a cadaveric dura mater graft. Case report. J Neurosurg.

[71] Thompson D, Taylor W, Hayward R (1994). Haemorrhage associated with silastic dural substitute. J Neurol Neurosurg Psychiatry.

[72] Costantino PD, Wolpoe ME, Govindaraj S, Chaplin JM, Sen C, Cohen M, Gnoy A (2000). Human dural replacement with acellular dermis: clinical results and a review of the literature. Head Neck.

[73] Persson C, Evans S, Marsh R, Summers JL, Hall RM (2010). Poisson’s ratio and strain rate dependency of the constitutive behavior of spinal dura mater. Ann Biomed Eng.

[74] Pierrat B, Carroll L, Merle F, Macmanus DB, Gaul R, Lally C, et al. Mechanical characterization and modeling of the porcine cerebral meninges. Front Bioeng Biotechnol. 2020;8:801. 10.3389/fbioe.2020.00801. 10.3389/fbioe.2020.00801 PMC7487364 32984262

[75] Sudres P, Evin M, Wagnac E, Bailly N, Diotalevi L, Melot A, Arnoux PJ, Petit Y (2021). Tensile mechanical properties of the cervical, thoracic and lumbar porcine spinal meninges. J Mech Behav Biomed Mater.

[76] Zwirner J, Ondruschka B, Scholze M, Schulze-Tanzil G, Hammer N (2019). Mechanical and morphological description of human acellular dura mater as a scaffold for surgical reconstruction. J Mech Behav Biomed Mater.

[77] Kriewall TJ, Akkas N, Bylski DI, Melvin JW, Work BA (1983). Mechanical behavior of fetal dura mater under large axisymmetric inflation. J Biomech Eng.

[78] Pei M, Zou D, Gao Y, Zhang J, Huang P, Wang J, Huang J, Li Z, Chen Y (2021). The influence of sample geometry and size on porcine aortic material properties from uniaxial tensile tests using custom-designed tissue cutters, clamps and molds. PLoS One.

