# Supplementary material for: Tensile properties of human spinal dura mater and pericranium
Source: J Mater Sci Mater Med. 2022 Dec 31;34(1):4. doi: 10.1007/s10856-022-06704-0 (PMC9805418; doi:10.1007/s10856-022-06704-0)
Supplement: Supplementary file 1 — Supplementary Materials [file 10856_2022_6704_MOESM1_ESM.docx]

# Supplementary Material

## A – Pilot tests

Pilot tests were conducted to identify the middle of the linear region for each region*orientation. Briefly, three samples for each group were tested in tension (Figure S1) until failure without preconditioning, and the average strain corresponding to the approximate middle of the quasi-linear region was determined for each group. A fourth sample for each group underwent cycles of sub-yield tensile loading at 1.6 mm/min and the stress-strain curves were stable (Figure S1) after three complete cycles of load. These pilot tests therefore established that three cycles of sub-yield tensile loading and unloading were sufficient for preconditioning.


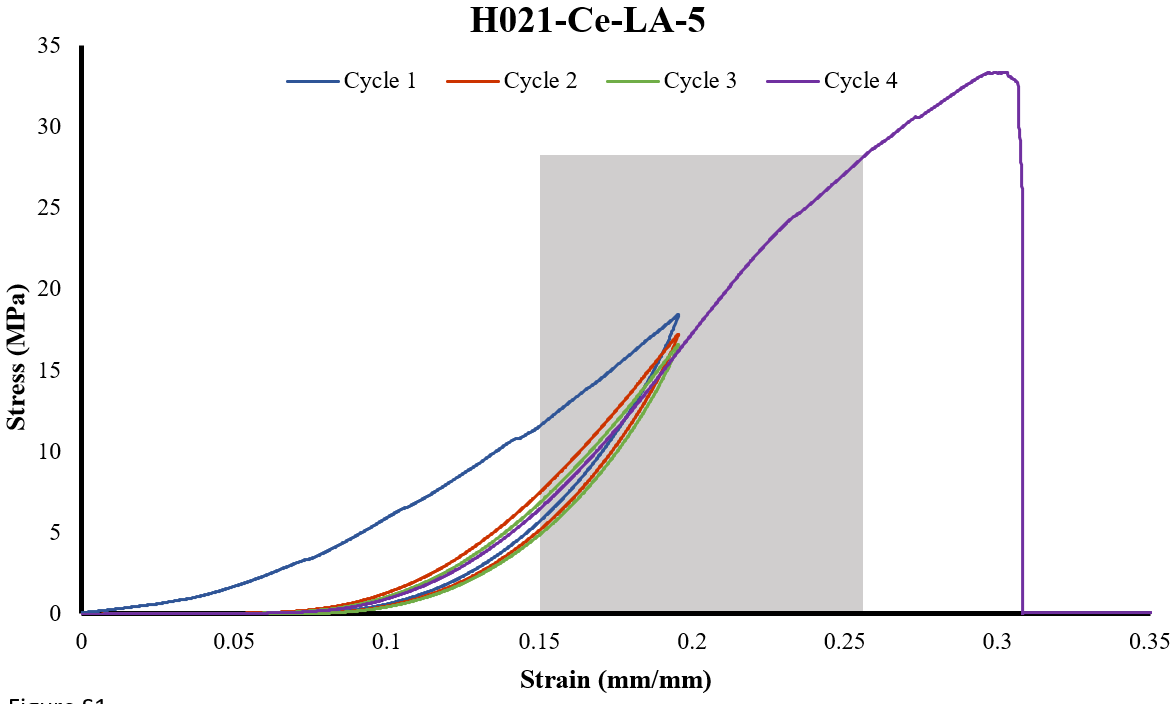


Figure S1: Stress-strain response during pre-conditioning, then load to failure, for H021-Ce-LA-5. The quasi-linear elastic region is indicated by the grey box. The response had stabilized by the third cycle of sub-yield loading.

## B – Ogden model fitting


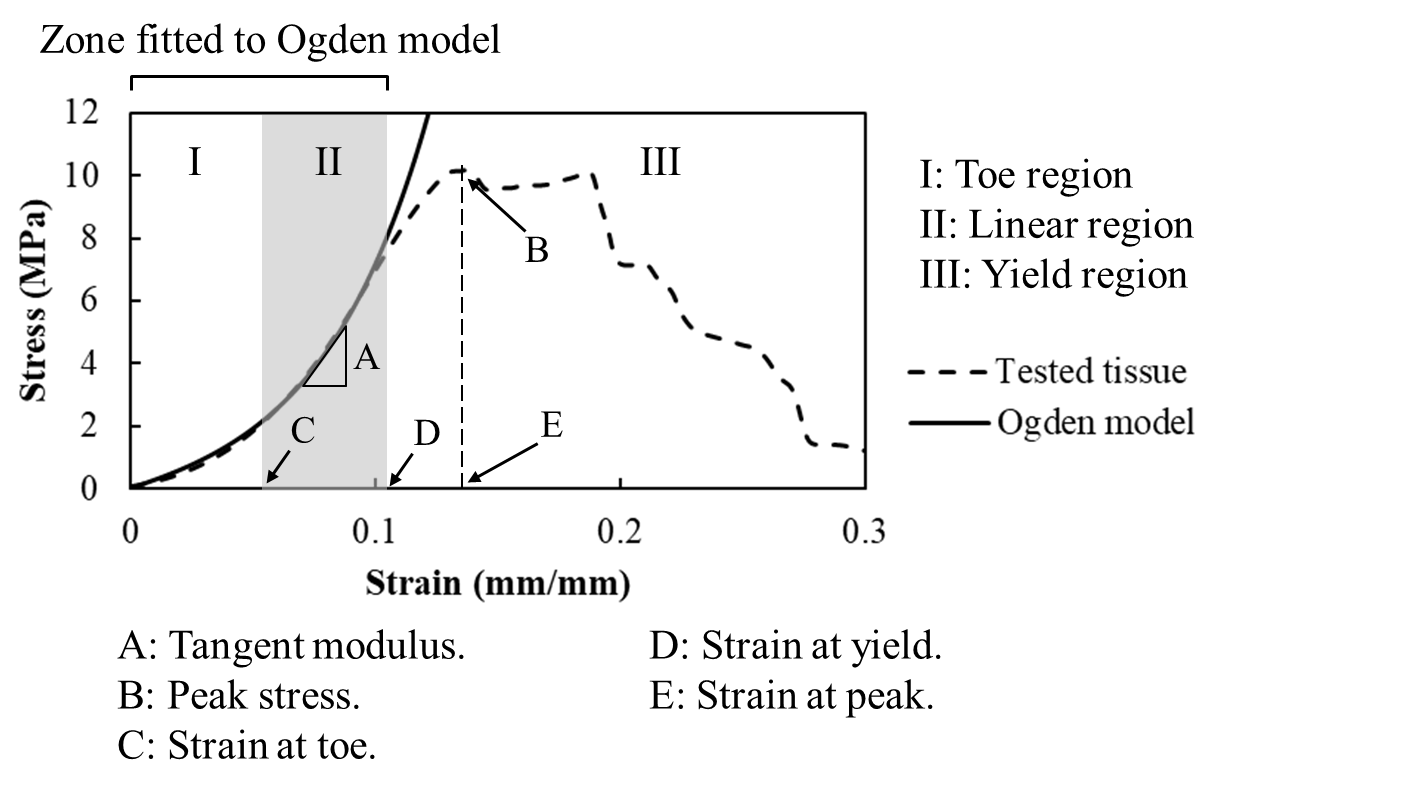


Figure S2: An example stress-strain curve and its Ogden model fit. The regions and outcomes are indicated.
